# Improving the User Interface and Guiding the Development of Effective Training Material for a Clinical Research Recruitment and Retention Dashboard: Usability Testing Study

**DOI:** 10.2196/66718

**Published:** 2025-02-24

**Authors:** Leah Leslie Gardner, Pezhman Raeisian Parvari, Mark Seidman, Richard J Holden, Nicole R Fowler, Ben L Zarzaur, Diana Summanwar, Cristina Barboi, Malaz Boustani

**Affiliations:** 1 Center for Health Innovation and Implementation Science, School of Medicine, Indiana University, 410 W 10th St #1140, Indianapolis, IN, 46202, United States, 1 (317) 274-8536; 2 Department of Medicine, School of Medicine, Indiana University, Indianapolis, IN, United States; 3 School of Public Health, Indiana University Bloomington, Bloomington, IN, United States; 4 Center for Aging Research, Regenstrief Institute Inc, Indianapolis, IN, United States; 5 Department of Surgery, Division of Acute Care and Regional General Surgery, University of Wisconsin-Madison, Madison, WI, United States; 6 Department of Family Medicine, School of Medicine, Indiana University, Indianapolis, IN, United States; 7 Department of Anesthesiology, School of Medicine, Indiana University, Indianapolis, IN, United States; 8 Sandra Eskenazi Center for Brain Care Innovation, Eskenazi Health, Indianapolis, IN, United States

**Keywords:** recruitment strategies, clinical research, research subject recruitment, agile science, agile implementation, human-computer interaction

## Abstract

**Background:**

Participant recruitment and retention are critical to the success of clinical trials, yet challenges such as low enrollment rates and high attrition remain ongoing obstacles. RecruitGPS is a scalable dashboard with integrated control charts to address these issues by providing real-time data monitoring and analysis, enabling researchers to better track and improve recruitment and retention.

**Objective:**

This study aims to identify the challenges and inefficiencies users encounter when interacting with the RecruitGPS dashboard. By identifying these issues, the study aims to inform strategies for improving the dashboard’s user interface and create targeted, effective instructional materials that address user needs.

**Methods:**

Twelve clinical researchers from the Midwest region of the United States provided feedback through a 10-minute, video-recorded usability test session, during which participants were instructed to explore the various tabs of the dashboard, identify challenges, and note features that worked well while thinking aloud. Following the video session, participants took a survey on which they answered System Usability Scale (SUS) questions, ease of navigation questions, and a Net Promoter Score (NPS) question.

**Results:**

A quantitative analysis of survey responses revealed an average SUS score of 61.46 (SD 23.80; median 66.25) points, indicating a need for improvement in the user interface. The NPS was 8, with 4 of 12 (33%) respondents classified as promoters and 3 of 12 (25%) as detractors, indicating a slightly positive satisfaction. When participants compared RecruitGPS to other recruitment and study management tools they had used, 8 of 12 (67%) of participants rated RecruitGPS as better or much better. Only 1 of 12 (8%) participants rated RecruitGPS as worse but not much worse. A qualitative analysis of participants’ interactions with the dashboard diagnosed a confusing part of the dashboard that could be eliminated or made optional and provided valuable insight for the development of instructional videos and documentation. Participants liked the dashboard’s data visualization capabilities, including intuitive graphs and trend tracking; progress indicators, such as color-coded status indicators and comparison metrics; and the overall dashboard’s layout and design, which consolidated relevant data on a single page. Users also valued the accuracy and real-time updates of data, especially the integration with external sources like Research Electronic Data Capture (REDCap).

**Conclusions:**

RecruitGPS demonstrates significant potential to improve the efficiency of clinical trials by providing researchers with real-time insights into participant recruitment and retention. This study offers valuable recommendations for targeted refinements to enhance the user experience and maximize the dashboard’s effectiveness. Additionally, it highlights navigation challenges that can be addressed through the development of clear and focused instructional videos.

## Introduction

Clinical studies are essential to advancing medical science and improving patient outcomes, and their successful completion relies on adequate participant recruitment [1]. Effective and efficient patient recruitment and retention ensure timely collection of data, while insufficient enrollment often leads to delays or even trial failure [2-5]. Studies indicate that 80% of trials face recruitment challenges [6], while 37% fail to meet their target sample size goals [7]. In fact, inadequate recruitment is the leading cause of trial discontinuation [8,9].

Clinical trial management systems (CTMSs), clinical trial recruitment support systems (CTRSSs), web-based platforms, patient registries, social media, and community engagement initiatives [10-14] are widely used to support recruitment and management of clinical trials. Electronic health records (EHRs) facilitate targeted recruitment by identifying potential participants based on health data, while CTMSs track participant enrollment, scheduling, and communication [15,16]. Online platforms like ClinicalTrials.gov provide global access to trial information, and social media platforms such as Facebook enable broader outreach through targeted advertisements [17,18]. While these tools offer valuable support for recruitment, they often face limitations such as high costs, limited reach, or sustainability challenges [19].

Dashboards are effective tools in clinical settings, particularly for tracking quality and safety metrics [20]. In clinical trials, dashboards are used to generate alerts when participant accrual is inadequate [3,21]. Commercially available CTMSs with dashboards to support large-scale clinical trials include Medidata Rave (Medidata Solutions) [22], Veeva Vault CDMS (Veeva Systems) [23], Ennov Clinical (Ennov) [24], Viedoc (Viedoc Technologies AB) [25], RealTime eClinical (RealTime Software Solutions) [26], and Clinevo (Clinevo Technologies) [27]. These systems have a variety of features for trial oversight, from patient recruitment to data monitoring, such as patient portals to streamline patient communication, consent capture, financial tracking, regulatory compliance, adverse event tracking, and study closeout.

Castor EDC (Castor) [28] and Ripple Science (Ripple Science Inc) [29] offer affordable packages for academic institutions and smaller companies. These packages have dashboards that help researchers track study progress, tasks, and participant data across different trials and that automate email or in-app notifications and reminders to both staff and participants.

OpenClinica (OpenClinica LLC) [30] and REDCap (Research Electronic Data Capture; Vanderbilt University) [31] are examples of open-source platforms for managing clinical trial data. OpenClinica Insight is an add-on module for OpenClinica 3 Enterprise and OpenClinica 4 that allows the user to create reports and dashboards. REDCap has limited dashboarding capabilities and requires the user to export data to a spreadsheet, such as Microsoft Excel, or statistical package, such as R and R Shiny, for detailed analysis and dashboard development.

Since REDCap requires the user to export data to a spreadsheet or other software for analysis, a research group from the Center for Health Innovation and Implementation Science (CHIIS) successfully developed and implemented an Excel-based dashboard in a single, multisite clinical study already in progress using REDCap [32]. This dashboard integrates control charts and enables seamless data updates from the REDCap database, allowing for effective monitoring of recruitment and participant progress. In this study, participants moved through various stages of the research workflow. The dashboard provided investigators with weekly updates on participant progress through these stages, facilitating timely interventions when participant counts or progress rates at any stage suggested that sample size targets might not be met. Additionally, it enabled the application of Agile science methods [33-37] to rapidly test and implement strategies for improving recruitment.

Building on the success of the dashboard used in the multisite clinical study, the research group developed a customizable, generic dashboard called RecruitGPS, which can be customized for other clinical research studies. This paper focuses on the detailed usability testing of the RecruitGPS user interface. A comprehensive description of RecruitGPS features is provided in Multimedia Appendix 1. The aim of this study is to identify the challenges and inefficiencies users experienced with the RecruitGPS interface so that these insights can be leveraged to improve usability and develop effective training materials.

## Methods

### Overview of the RecruitGPS Dashboard

**Figure 1.**
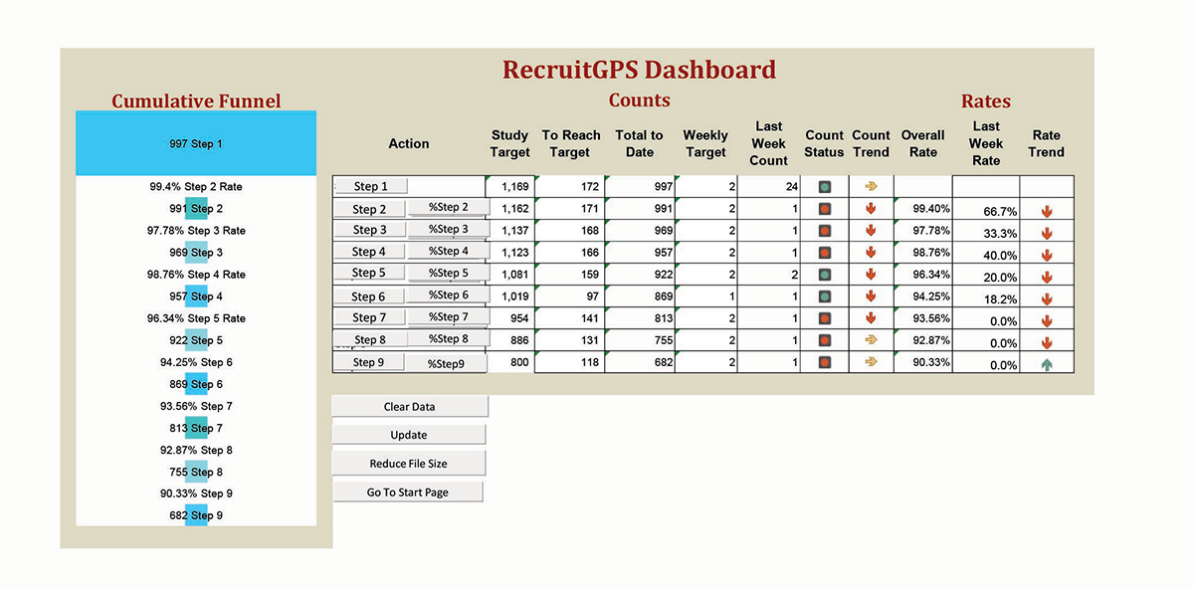
The RecruitGPS dashboard interface evaluated during usability testing.

The goal of the user interface is to provide clinical trial principal investigators and research coordinators with immediate access to weekly and cumulative counts at each stage of the study and weekly and cumulative transition rates from stage to stage. The interface uses compelling visual cues to bring the user’s attention to potential problems in the study workflow. The first version of the user interface for RecruitGPS seen in Figure 1 was the version from the successful multisite study dashboard user interface [32] with the generic names step 1 through step 9 substituted for the names of the stages screened through 18-month assessment. The multisite study user interface was collaboratively designed by the stakeholders in the study and the dashboard developer.

Think-aloud usability testing sessions were combined with a user survey to assess the effectiveness of, efficiency of, and satisfaction with the RecruitGPS dashboard. Effectiveness is the extent to which the user can achieve a goal with accuracy and completeness. Efficiency is the effort and resources necessary to achieve a complete and accurate goal. Satisfaction is a positive user experience and absence of discontent during task performance. Once the 3 are fulfilled adequately, the product can be considered to have attained an acceptable level of usability [38].

### Participant Recruitment

Participants in the study were recruited to use the RecruitGPS user interface while being video recorded and to complete a survey about their experience. Most participants were affiliated with CHIIS or were clinical researchers primarily based in the Midwest region of the United States. Recruitment and usability testing were conducted in August 2024.

Participants were recruited using convenience and snowball sampling techniques, with the CHIIS team supporting the process through digital advertisements, emails, and word of mouth. To be eligible, participants needed experience with clinical research studies, fluency in English, and access to the internet and Zoom (Zoom Video Communications Inc.). Although the literature suggests that 5 to 8 participants are sufficient for think-aloud usability testing, 12 participants were recruited to ensure diverse and comprehensive insights [39,40].

### Usability Testing Sessions and Surveys

A total of twelve 10-minute usability testing sessions of the RecruitGPS dashboard were conducted via Zoom. During each session, the facilitator shared their screen, displaying the dashboard, and participants were provided with remote control access to interact with the interface. Participants were instructed as follows: “consider[ing] a research project of your own, explore the various tabs of the dashboard, identify challenges, and note features that work well while thinking aloud.” To identify specific problems, the researchers recorded the 10-minute usability testing sessions for a qualitative analysis of the challenges users encountered and features that worked well.

Following each usability test, participants received a survey link to provide additional feedback. The survey gathered insights on users’ overall experience, including their System Usability Scale (SUS) (Textbox 1) [41] and Net Promoter Score (NPS) [42] ratings. The NPS is based on the question “On a scale of 0 to 10, how likely are you to recommend this dashboard to a colleague or friend?” It is reported as a number ranging from −100 to +100, where a higher score is desirable. Respondents are categorized as detractors (giving scores of 0‐6), passives (giving scores of 7‐8), and promoters (giving scores of 9‐10) The percentages of promoters and detractors are calculated and then the percentage of detractors is subtracted from the percentage of promoters to obtain the NPS [42]. The survey also included open-ended questions about the most and least useful features, as well as overall impressions of the user experience and dashboard interface. To assess ease of navigation in the survey, users were asked the questions in Table 1.

Textbox 1. **System Usability Scale (SUS) questions in the survey following the usability test.**

**SUS Questions (1=strongly disagree, 2=disagree, 3=neutral, 4=agree, 5=strongly agree)**

1. I think that I would like to use this system frequently.
2. I found the system unnecessarily complex.
3. I thought the system was easy to use.
4. I think that I would need the support of a technical person to be able to use this system.
5. I found the various functions in this system were well integrated.
6. I thought there was too much inconsistency in this system.
7. I would imagine that most people would learn to use this system very quickly.
8. I found the system very cumbersome to use.
9. I felt very confident using the system.
10. I needed to learn a lot of things before I could get going with this system.

**Table 1.** Questions on ease of navigation in the survey following the usability test.

Question	Available responses
How easy was it to navigate through the dashboard?	Likert scale (1‐5, with 1 being the most difficult and 5 being the easiest)
Overall, how does this recruitment dashboard compare to other recruitment tools you have used?	Scale: 1=much worse, 2=worse, 3=about the same, 4=better, 5=much better
How intuitive did you find the user interface (UI) of this recruitment dashboard?	Scale: 1=not intuitive at all, 2=slightly intuitive, 3=neutral, 4=intuitive, 5=very intuitive

### Ethical Considerations

This study was approved by the Human Research Protection Program at Indiana University (23755) prior to participant recruitment. Informed consent was obtained from all participants. Throughout the study, strict confidentiality and privacy protocols were followed. All data collected were deidentified before analysis to maintain participant anonymity. To protect privacy, secure data storage and anonymization procedures were used. Furthermore, no identifiable images of participants were included in the data. Participation was voluntary and no financial compensation or incentives were provided.

## Results

This analysis assesses the effectiveness and efficiency of the RecruitGPS dashboard and satisfaction with it.

### Participants

Twelve medical professionals completed the usability testing and surveys. Their demographics are summarized in Table 2.

**Table 2.** Education levels, roles, and experience of study participants (N=12) completing the usability testing and surveys.

Characteristics	Participants, n (%)
**Education**	
Doctorate (PhD)	2 (17)
Master’s degree	4 (33)
Professional degree (MD, JD)	6 (50)
**Role in research**	
Principal investigator or coprincipal investigator	5 (42)
Project manager	5 (42)
Research coordinator	2 (17)
**Experience**	
1‐3 years	4 (33)
4‐6 years	2 (17)
7‐10 years	1 (8)
Less than 1 year	1 (8)
More than 10 years	4 (33)

### Effectiveness

The 10 questions on the SUS together assess overall usability, computed on a scale from 0 to 100, as described in detail by Brooke [41]. Table 3 reports individual item scores and overall SUS scores for each participant. Higher ratings on odd-numbered questions and lower ratings on even-numbered questions indicate better usability.

**Table 3.** System Usability Scale (SUS) total scores and scores for each question (Q) by participant (P).

	P1	P2	P3	P4	P5	P6	P7	P8	P9	P10	P11	P12
Q1	2	3	2	4	3	3	4	1	4	4	3	4
Q2	1	4	1	1	2	3	3	1	2	1	3	4
Q3	1	4	1	3	3	3	3	0	3	3	3	4
Q4	1	4	0	2	3	0	3	0	1	3	3	4
Q5	2	3	2	3	3	3	2	1	2	3	3	4
Q6	4	4	2	2	3	3	3	1	4	3	3	4
Q7	1	3	2	4	2	3	3	0	3	3	3	4
Q8	2	4	1	4	2	3	3	1	1	3	3	4
Q9	0	2	0	3	2	3	2	0	1	3	3	4
Q10	1	1	0	4	2	1	2	1	4	2	3	4
SUS score	37.5	80.0	27.5	75.0	62.5	62.5	70.0	15.0	62.5	70.0	75.0	100.0

The mean SUS total score for all participants was 61.46 (SD 23.80; median 66.25) points. According to Bangor et al [43], a score of 85 and above is excellent and a score of 70‐84 is good.

### Efficiency—Assessing Ease of Navigation

Feedback on ease of navigation showed a mixed picture. While 25% (n=3) of the 12 respondents gave a rating of 5% and 58% (n=7) gave a rating of 4, 17% (n=2) rated ease of navigation a 1.

### Satisfaction

The NPS [42] for RecruitGPS in this study was 8, with 33% (n=4) of respondents classified as promoters and 25% (n=3) as detractors. This indicates a slightly positive satisfaction.

A second question asked participants to compare RecruitGPS to other recruitment tools they had used on a scale of 1‐5, with 1 meaning RecruitGPS is much worse and 5 meaning Recruit GPS is much better. The results were that 8 of 12 participants thought RecruitGPS was better. The results are summarized in Table 4.

**Table 4.** Survey responses (N=12) comparing RecruitGPS to other recruitment tools used by participants.

Score	Interpretation	Number of responses, n (%)
1	Much worse	0 (0)
2	Worse	1 (8)
3	About the same	3 (25)
4	Better	5 (42)
5	Much better	3 (25)

### Qualitative Feedback From Participants

The framework developed by Clarke and Braun [44] was used for thematic analysis of the recorded 10-minute usability testing sessions to identify and analyze patterns within the data, including challenges users encountered and features that worked well.

#### Challenges

Several users reported difficulties with specific graphical elements, such as the cumulative funnel, which they found confusing or unintuitive. Additionally, some users experienced challenges with unclear instructions and labels, leading to difficulties in interpreting data and understanding the dashboard’s functionality. These issues were reflected in the variability of ease of navigation ratings, with some users finding the dashboard extremely difficult to use.

#### Features That Worked Well

Positive feedback centered on the dashboard’s data visualization capabilities, including intuitive graphs and trend tracking, which were highly valued by users. Progress indicators, such as color-coded status indicators and comparison metrics, were appreciated for helping users monitor their recruitment goals effectively. The dashboard’s layout and design, which consolidated relevant data on a single page with clear button labeling, contributed to user satisfaction. Users also valued the accuracy and real-time updates of data, especially the integration with external sources like REDCap, which was seen as a critical feature for effective data management.

## Discussion

### Principal Findings

We found that 8 of 12 (67%) participants considered RecruitGPS better than other recruitment tools they had used, but the usability (SUS), satisfaction (NPS), and efficiency scores all indicated a need for improvement. Feedback on ease of navigation showed significant efficiency challenges faced by a minority of users, suggesting that some users struggled with navigation, despite overall positive feedback.

The recommendations for improvement based on the qualitative analysis are follows:

1. Remove the cumulative funnel or make it an optional feature for users who request it.
2. Create videos with accompanying documentation to address challenges users encountered with (a) instructions and labels and (b) navigation.

Although the quantitative analysis highlighted areas for improvement, the testing was conducted without any prior training for participants. The developers of RecruitGPS had not anticipated users attempting to operate the system without access to documentation or instructional materials, such as training videos. Despite this, RecruitGPS performed well enough. Additionally, the qualitative analysis offered valuable insights for enhancing the documentation and provided clear guidance for the development of instructional videos.

### Limitations

The first limitation of this study was the relatively small sample size, consisting of only 12 participants in the usability testing sessions and survey responses. Conducting future studies with a larger and more diverse user group could yield additional insights and a broader understanding of user needs.

A related limitation was the participants’ affiliations, as most were connected to 1 of 2 medical schools or their associated health care systems. While some participants had experience at other research institutions, their familiarity with alternative recruitment tools may have been limited to those used within these 2 medical schools, potentially restricting the study’s generalizability.

Another limitation was that the study did not include testing of the existing documentation, and the instructional videos had not yet been developed. Future testing of these resources could provide valuable feedback to refine and improve their effectiveness.

### Conclusions

RecruitGPS demonstrates promise and potential in enhancing recruitment and retention for clinical trials by providing real-time insights, supporting agile decision-making, and using scalable design with an accessible user interface. Removing the cumulative funnel and creating instructional videos and documentation focused on navigation challenges are likely to enhance its usability. With these enhancements, RecruitGPS could become a key tool in clinical trial management, supporting successful participant recruitment and better research outcomes.

## Supplementary material

10.2196/66718 Multimedia Appendix 1 Detailed features of RecruitGPS dashboard.

## Abbreviations

CHIIS: Center for Health Innovation and Implementation Science
CTMS: clinical trial management system
CTRSS: clinical trial recruitment support system
EHR: electronic health record
NPS: Net Promoter Score
REDCap: Research Electronic Data Capture; Vanderbilt University
SUS: System Usability Scale
