# Supplementary material for: Improving the User Interface and Guiding the Development of Effective Training Material for a Clinical Research Recruitment and Retention Dashboard: Usability Testing Study
Source: JMIR Form Res. 2025 Feb 24;9:e66718. doi: 10.2196/66718 (PMC11875103; doi:10.2196/66718)
Supplement: Multimedia Appendix 1 [file formative-v9-e66718-s001.doc]

## **Appendix 1: Detailed Features of RecruitGPS Dashboard**

### **What is a RecruitGPS?**

The primary purpose of the dashboard with control charts is to provide the principal investigators and other stakeholders of clinical research studies with weekly updates on the number of participants who have completed each stage or step within the process, both for the current week and cumulatively. This dashboard is linked to control charts enabling trend analysis for the timely identification of recruitment-related issues and allowing users to delve into time-series data for thorough intervention evaluation. This dashboard can be used to analyze any staged process with attrition, such as admissions management in educational programs and employee recruitment.

### **Overview**

The Start Page allows users to select from three main dashboards by clicking on the buttons as shown below:


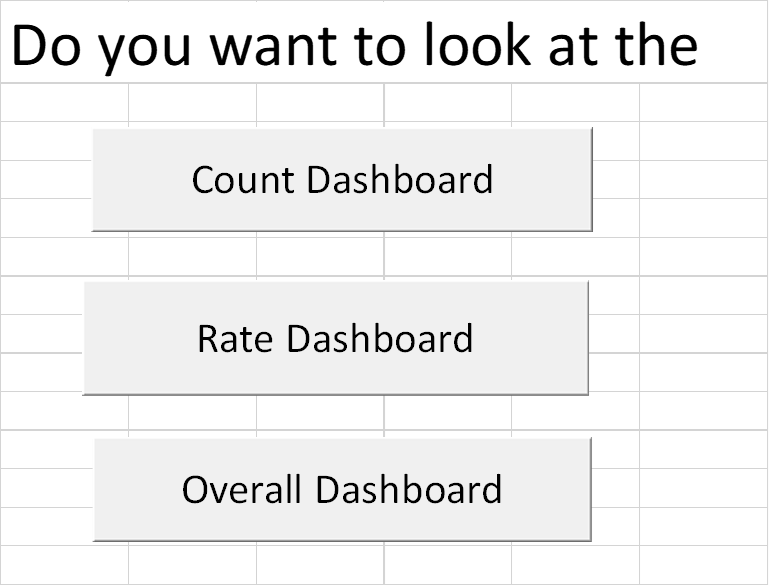


The Count Dashboard shows only count data from the Overall Dashboard and the Rate Dashboard shows only Rate Data from the Overall Dashboard. The Overall Dashboard shows both Count and Rate Data and is shown below. Each Dashboard has navigation buttons to take the user to the respective control chart worksheets and back to the Start Page. Each Dashboard also has buttons for calling macros to clear old data and update all worksheets when new data has been pasted onto the Generic Data Worksheet.


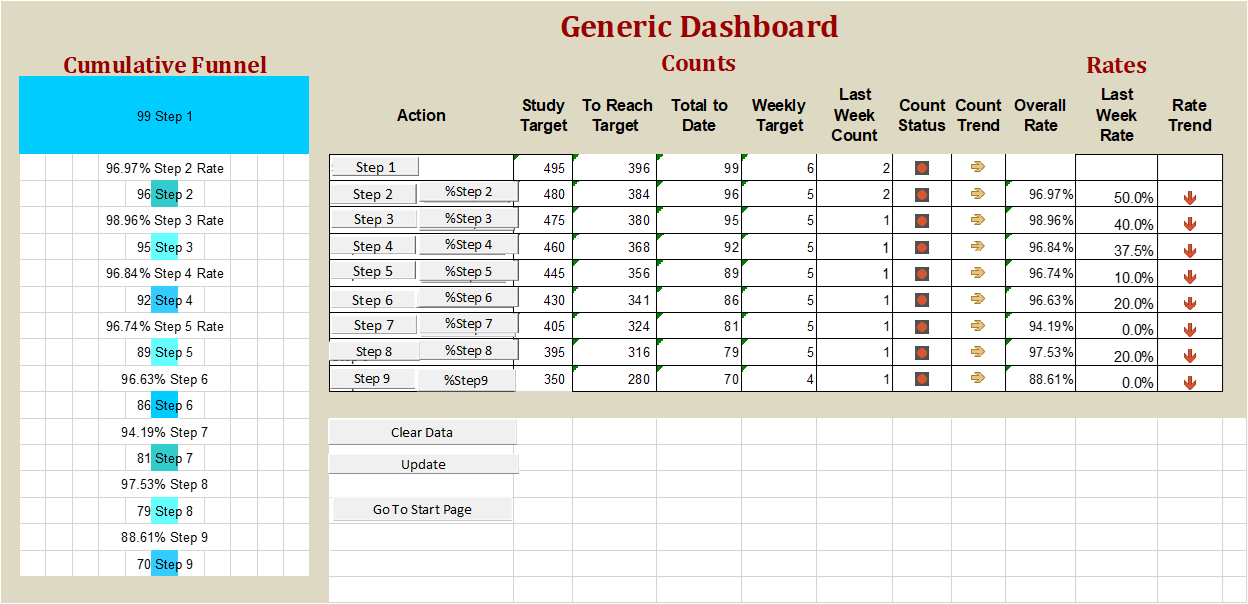


The cumulative funnel shows how the numbers of patients decline through the various stages in the screening and consent process.

The **Action column** of the worksheet contains descriptions of the steps or stages of the process. The Study Target column contains the number of patients needed to reach a study goal at the Baseline level based on the rates and cumulative rates at each stage given in the Rate and Cum.

**Rate columns** values can change as the rates change. The **To Reach Target column** contains the number of patients who must be processed in the future at each stage required to reach the goal Baselines. These values also change as the rates change and as more patients are processed. The **Weekly Target column** gives the number in **To Reach Target** divided by the number of weeks remaining. The **Total to Date** column tells the total number of patients processed at each stage to the end of the most recent full week.

Except for the Withdrawn row, the **Count Status** column contains a stoplight that is:

- Green if the **Last Week Count** is within 10% of target value,
- Yellow if the **Last Week Count** value is between 70% and 90% of target value,
- Red if the last value is less than 70% of target value.

This column contains a formula for the difference between the current month and the target value. It is conditionally formatted according to the rule for stoplight colors. The withdrawn column gives a green light if there are no withdrawals, a yellow light for less than target but more than none, and a red light for more than target in the most recent week.

The **Count Trend** column compares the average count of the last four weeks to the **Weekly Average** count. The symbols are:

-
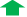
If the average of the last 4 weeks is 2 standard deviations or more above overall mean.
-
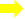
If the average of the last 4 weeks is within 2 standard deviations of overall mean.
-
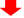
If the average of the last 4 weeks is 2 standard deviations or more below overall mean.

The **Overall Rate** column is the number of patients at the current stage divided by the number at the preceding stage. The **Last Week Rate** column contains the rate for the most recent full week. in the date respectively. These metrics are taken from the related control charts and the cells contain formulas linking them to the appropriate control chart cells.

The **Rate Trend** column compares the average rate of the last four weeks to the **Weekly Average** rate. The symbols are:

-
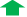
If the average of the last 4 weeks is 2 standard deviations or more above overall rate.
-
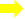
If the average of the last 4 weeks is within 2 standard deviations of overall rate.
-
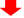
If the average of the last 4 weeks is 2 standard deviations or more below overall rate.

**Control Chart Worksheets**

This workbook contains a control chart worksheet for each Action count and for each Action rate except for which a rate exists (rate does not exist for Screened because there is no preceding stage). These are accessible using the navigation buttons in the Action column or by paging across the tabs at the bottom of the worksheet.

A control chart is a time plot of data from a process for the purpose discerning whether the process is stable, with variation only coming from sources common to the process, or not stable, with variation coming from identifiable causes. Control charts are used to identify patterns of variation that are statistically significant, so the user does not react to variation that is not statistically significant.


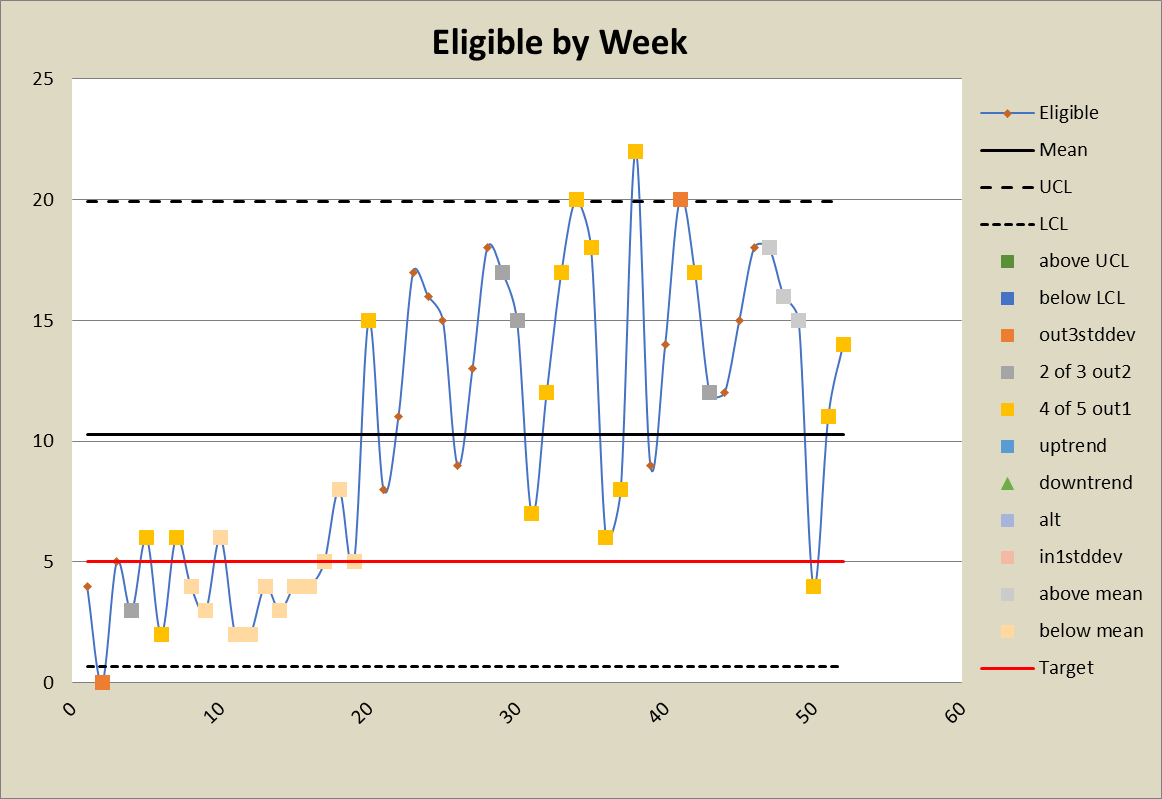


**Data Requirements**

The data to be pasted into the Generic Data sheet should be of the format shown below with a text participant ID of any format as long as it is text, followed by a date in the mm/dd/yyyy format for each step and the withdrawal date if the participant withdraws. If a participant withdraws, the dates for steps following the withdrawal date should be 0 or 1/0/1900. If a participant does not withdraw, the withdrawal date should be 0 or 1/0/1900.

| Patient ID | Step 1 Date | Step 2 Date | Step 3 Date | Step 4 Date | Step 5 Date | Step 6 Date | Step 7 Date | Step 8 Date | Step 9 Date | Withdrew Date |
| --- | --- | --- | --- | --- | --- | --- | --- | --- | --- | --- |
| p1 | 6/29/2023 | 7/18/2023 | 9/13/2023 | 10/13/2023 | 11/6/2023 | 2/9/2024 | 4/5/2024 | 5/15/2024 | 6/30/2024 | 1/0/1900 |
| p2 | 11/22/2023 | 12/12/2023 | 1/2/2024 | 2/10/2024 | 3/25/2024 | 1/0/1900 | 1/0/1900 | 1/0/1900 | 1/0/1900 | 4/11/2024 |
| p3 | 11/20/2023 | 12/1/2023 | 12/14/2023 | 1/7/2024 | 2/6/2024 | 3/18/2024 | 5/25/2024 | 6/30/2024 | 8/26/2024 | 1/0/1900 |
| p4 | 10/24/2023 | 10/30/2023 | 12/1/2023 | 12/23/2023 | 1/16/2024 | 2/21/2024 | 3/29/2024 | 5/16/2024 | 7/17/2024 | 1/0/1900 |
| p5 | 4/20/2023 | 4/26/2023 | 6/4/2023 | 7/12/2023 | 9/2/2023 | 9/27/2023 | 1/10/2024 | 3/10/2024 | 7/29/2024 | 1/0/1900 |
| p6 | 8/27/2023 | 9/16/2023 | 9/27/2023 | 10/17/2023 | 11/16/2023 | 1/8/2024 | 2/15/2024 | 4/30/2024 | 6/16/2024 | 1/0/1900 |
| p7 | 10/16/2023 | 10/25/2023 | 11/4/2023 | 12/17/2023 | 1/6/2024 | 2/16/2024 | 4/23/2024 | 5/31/2024 | 7/24/2024 | 1/0/1900 |
| p8 | 1/9/2024 | 1/24/2024 | 2/6/2024 | 3/15/2024 | 4/19/2024 | 6/12/2024 | 11/14/2024 | 12/24/2024 | 5/14/2025 | 1/0/1900 |
| p9 | 9/20/2023 | 10/3/2023 | 10/15/2023 | 11/27/2023 | 12/20/2023 | 3/4/2024 | 6/18/2024 | 8/16/2024 | 11/12/2024 | 1/0/1900 |
| p10 | 1/8/2024 | 1/14/2024 | 2/14/2024 | 4/10/2024 | 5/2/2024 | 6/21/2024 | 7/26/2024 | 9/10/2024 | 11/26/2024 | 1/0/1900 |
